# Density- and Size-Dependent Winter Mortality and Growth of Late *Chaoborus flavicans* Larvae

**DOI:** 10.1371/journal.pone.0075839

**Published:** 2013-10-04

**Authors:** Arne Schröder

**Affiliations:** Department of Animal and Plant Sciences, University of Sheffield, Sheffield, United Kingdom; University of Connecticut, United States of America

## Abstract

Winter processes such as overwinter survival and growth of individuals can have wide-ranging consequences for population dynamics and communities within and across seasons. In freshwater organisms winter processes have been mainly studied in fish despite that invertebrates also have substantial impacts on lake and pond food webs. One of the major invertebrate consumers in lake and ponds is the planktonic larvae of the dipteran insect *Chaoborus* spec. However, while much is known about *Chaoborus* feeding ecology, behaviour and structuring role in food webs, its winter ecology and how it affects its populations are poorly understood. Here size- and density-dependent winter mortality and body growth of late *Chaoborus flavicans* larvae were quantified over naturally occurring size and density ranges in autumn and under natural winter conditions using two field enclosure experiments. Winter mortality increased with autumn density but decreased with autumn body size while winter growth rates decreased with autumn density and body sizes. There was also a density- and size-independent background mortality component. The proportion of pupae found in spring decreased strongly and exponentially with autumn density. These results may explain the commonly observed univoltine life cycle and multi-annual density fluctuations in northern *Chaoborus* populations. They further demonstrate the relevance of winter processes and conditions for freshwater invertebrates and ecosystems.

## Introduction

A substantial fraction of the Earth’s lakes and ponds are found in temperate and boreal regions [1] with their pronounced seasonality. Environmental conditions in such lakes and ponds vary greatly over a year and are usually highly adverse during winters which are times of food shortage, low oxygen levels, minimum temperature and low light intensity; especially when lakes and ponds are covered with ice [2], [3], [4]. Freshwater organisms have evolved many physiological, behavioural and life history strategies to deal with winter conditions. But despite that these adaptations generally involve reduced activity or energetic requirements, diapause stages or seasonal patterns in development and reproduction winters are not simply times of dormancy without any fitness or dynamical consequences. Winter conditions especially influence an individual’s winter survival chance or its winter loss/gain rates of biomass through starvation or growth. These processes are, because of size-specific resource use, physiological demand and energy reserve mobilisation [5], density- and size-dependent. These density- and size-dependent processes in turn can affect population dynamics, density and size structure within and across seasons directly but also indirectly through density-dependent regulatory feedback loops [6], [7]. Moreover, effects of winter survival and growth may also be transferred via trophic interactions to the whole lake food web.

For example, in fish populations size- and density-dependent winter mortality can influence density and size structure in summer and autumn through size- and density-dependent year class survival, growth rates and/or energy accumulation all which in turn can influence winter mortality [8], [9], [10] and population dynamics [6], [7]. Reduction in fish biomass through winter mass kill-offs triggered by anoxia in the water column of ice-covered lakes can initiate trophic cascades, influencing the biomass and size distribution of lower trophic levels in summer [11], [12]. Planktivorous fish often avoid periods of low food availability but high predation pressure from piscivores in lakes by migrating into stream inlets over winter [13]. This winter migration can affect summer food web dynamics through the timing of the return migration and start of high consumption pressure on zooplankton grazers in spring [14].

It is thus evident that winter processes are of importance both in regulating populations of freshwater organisms and in shaping lake and pond food webs. However, winter growth and survival have predominantly been studied in fish [6], [7], [8], [9], [10] which are not the only freshwater consumers: invertebrates such as predatory crustaceans or many insect larvae have similarly wide-ranging top-down impacts on species composition, relative abundances and community size structure and thus on food web stability and dynamics [15], [16], [17], [18]. Further, because of their smaller body sizes, their reliance on chemical or haptic prey cues and their complex life cycles often involving shifts between terrestrial and aquatic habitats invertebrate consumers influence food webs differently than fish [19], [20], [21], [22].

One of the major invertebrate consumers in lakes and ponds are the larvae of the dipteran insect *Chaoborus* spec. or phantom midge. Especially its later larval stages can substantially supress herbivorous cladoceran zooplankton and thereby influence mesozooplankton community composition, dynamics and size structure (e.g. [15], [21], [22], [23], [24]). Their ontogenetic resource niche shift from mainly consuming microzooplankton such as rotifers during early larval stages towards feeding on mesozooplankton, especially cladocerans, during later stages [25], [26] can lead to alternative stable food web states [22], [27], [28]. *Chaoborus* feeding ecology (e.g. [25], [26], [29], [30]), their vertical migration behaviour (e.g. [31], [32]) and their species-specific interactions with planktivorous fish that prey on them (e.g. [33], [34]) have been widely studied. Nonetheless, despite the demonstrated impact of *Chaoborus* on lake food webs and the attention its ecology received, relatively little is known about the density- and size-dependent processes that regulate its population dynamics and especially little about its winter ecology.

In boreal and temperate regions *Chaoborus* species overwinter as larvae (e.g. [22]) with active metabolism [35]. They can be found in the water column under the ice [36] (A. Schröder personal observation) and they metamorphose into non-feeding pupae quickly in spring [37]. It can therefore be surmised that winter mortality and growth are important processes in *Chaoborus* populations. Here these processes were quantified for late *Chaoborus flavicans* larvae over naturally occurring autumn size and density ranges and under natural winter conditions using two field enclosure experiments. It is shown that winter mortality increases with autumn density but decreases with autumn body size while winter growth rates decreases with autumn density and with autumn body sizes.

## Materials and Methods

### Natural history

The experiments were carried out during the winters 2010/2011 (density dependence experiment; Exp. I) and 2011/2012 (size dependence experiment; Exp. II) in a small fishless and unproductive bog lake in central Sweden. The lake is located at 64°29′North, 19°26′East, has an area of 0.42 ha, a maximal depth of 7 m and a mean depth of 4 m. The water pH is 6.9 and the concentration of total phosphorus and total nitrogen is 8.3 and 907 µg L^−1^, respectively. Åman’s Fish Co-Operative allowed exclusive use of the experimental lake. The ground-water fed lake is situated in a sandy area, surrounded by pine forests mainly consisting of *Pinus silvestris* with reindeer lichens *Cladonia rangiferina* and lingon berries *Vaccinium vitis-idaéa* as the main ground vegetation. It freezes completely over between late-September/early-October and mid- to end-May. Dominant species in the pelagic food web are the herbivorous cladocerans *Holopedium*, *Bosmina* and *Ceriodaphnia* and the rotifer genera *Ascomorpha, Polyarthra, Keratella, Kellikotia* and *Conochilus*. Key predators in the lake are larvae of the dipteran *Chaoborus flavicans* MEIGEN (Diptera: Chaoboridae) [22]. In northern Europe, *Chaoborus* has a univoltine life cycle with non-overlapping generations. The short-living adults emerge from pupae and lay eggs in late spring/early summer. The larvae grow through three larval stages (instars I – III) to the fourth and final larval stage (instars IV) during summer, overwinter as such and pupate in spring [37]. Early instars feed on microzoplankton, especially rotifers, while later instars feed on mesozooplankton, predominantly small-bodied cladocerans [25], [26]. Pupae and adults do not feed.

### Enclosure set-up

Density-and size-dependent winter mortality and growth of *Chaoborus* larvae were assessed in lake enclosures. For each of the two experiments twelve plastic bags were filled with lake water in mid-September, shortly before the lake freezes over. The plastic bags had a diameter of 1.6 m and a depth of 4 m and were hanging in the lake water column supported by floating wooden frames anchored to the lake bottom. The water was filtered through a 200 µm cloth to prevent uncontrolled introductions of *Chaoborus* larvae and other possibly occurring large predatory invertebrates while establishing ambient autumn mesozooplankton densities in the enclosures. There were no systematic differences in cladoceran density (mainly *Bosmina* and *Chydorus)* between treatments in each experiment, which this late in the season was very low. In Exp. I, mean cladoceran density and its SD was 0.31 µg L^−1^±0.14 and the Spearman’s correlation coefficient between stocked *Chaoborus* and cladoceran density including its bootstrapped 95% CI equalled 0.02 (−0.48–0.47). In Exp. II, mean cladoceran density and its SD was 0.23 µg L^−1^±0.07 and did not differ between size treatments (ANOVA, F_3,8_ = 0.418, p = 0.745). Directly after filling, the enclosures were stocked with *Chaoborus* larvae which were obtained from twelve summer enclosures of the same design and placed in the same lake as the experimental winter enclosures. Summer enclosures were filled with lake water filtered through 70 µm cloth to reduce initial cladoceran densities to a minimum. Cladocerans suppress rotifers without which early *Chaoborus* instars cannot survive, leading to a recruitment failure [22], [38]. The summer enclosures were set out in late May each year and adults were allowed to naturally deposit eggs into them. In each September just prior to the start of the experiment summer enclosures were emptied of larvae with repeated vertical hauls of a net with 250 µm mesh size. Cultivating experimental individuals in enclosures instead of taking them from lakes where they may have encountered more natural conditions may have biased the results. However, cultivated larvae, pooled across both experiments, were in September on average 9.71 mm (9.57–9.81) long and thus of similar body size as larvae found in autumn lake samples (9.51 mm; 9.35–9.67), indicating only, if any, minor cultivation artefacts.

### Experimental design and sampling

For the density dependence experiment (Exp. I) *Chaoborus* larvae were introduced to the twelve winter enclosures on September 16^th^ 2010 with an increasing density of 7.5, 10.0, 12.5, 17.5, 22.5, 32.5, 42.5, 52.5, 62.5, 72.5, 82.5 and 127.5 *Chaoborus* larvae m^−3^. This range spans the autumn densities seen in this and an adjacent similar lake (0–131 larvae m^−3^) [22]. Larvae were randomly selected one-by-one for the first 6 lower density treatments and five-by-five for the 6 remaining higher density treatments and sorted into twelve buckets which were then gently emptied into randomly assigned enclosures. Before this sorting, a random subsample was taken from all larvae obtained from the summer enclosures in order to estimate the initial autumn size distribution, mean size and the percentage of different larval stages for all density treatments. The larvae of this subsample were counted using a hand counter and then digitally photographed together with a metal piece of known dimensions in a glass dish. Thus, body sizes of each individual could be obtained from measurements on a computer screen, measured as total length from the base of the antennae to the start of the anal paddle. Larval stage was determined for at least 50 individuals (all if more in a sample) by the length of the sclerotized head capsule, which does only change discreetly with moulting between larval stages, measured from the base of the antennae to the end of the distal end of the lateral capsule plate using a stereo microscope. The mean size in autumn was 11.21 mm ±1.61 SD with a bootstrapped 95% CI of 10.87–11.53 and all larvae were instars IV. On May 19^th^ and 20^th^ 2011, ca. 8–9 days after ice break, surviving individuals were recovered. Each enclosure was repeatedly sampled with vertical hauls of a net with 250 µm mesh size until no larvae was found in at least 10 hauls of which 3 had to be consecutively empty. Because of the size and homogeneity of the enclosures and the rigorous sampling regime I am confident to have retrieved virtually all surviving larvae despite that sampling occurred at daytime and the species can show daily vertical migration [22]. Samples were immediately preserved in Lugol’s solution. Recovered individuals were staged, counted, digitally photographed and their total lengths measured as described above.

For the size dependence experiment (Exp. II) each of the twelve winter enclosures received 31.25 larvae m^−3^ on September 22^nd^ 2011. Larvae obtained again from summer enclosures were first sorted visually into the four size classes Small (“S”), Medium (“M”), Large (“L”) and Extra-large (“XL”) and then randomly sorted five-by-five into twelve buckets, each of which was then randomly assigned to an enclosure and gently emptied into it. For each size class a random subsample was preserved to estimate the initial autumn size distribution, mean size and percentage of larval stages for all three replicates in this size class. In autumn, larvae in size class S were on average 6.82 mm long (SD ±0.94; 95% CI = 6.62–7.01), in size class M 9.06 mm long (SD ±0.78; 95% CI = 8.94–9.18), in size class L 9.97 mm long (SD ±0.73; 95% CI = 9.83–10.11) and in size class XL 11.53 mm long (SD ±0.91; 95% CI = 11.37–11.71). Larvae in size class S in autumn were to 70% instars III and to 30% instars IV, in size class M this was 18% and 72%, respectively, and size classes L and XL contained only instars IV. On May 23^rd^ 2012, not more than 3 days after ice break, surviving larvae were recovered, counted, staged and measured following the same procedures as in Exp. I.

### Calculations and data analysis

#### Mortality

For each enclosure of the density or size treatments the per-capita mortality M was calculated as 1–N_t+1_/N_t_ where N_t_ is the stocked density in autumn and N_t+1_ is the recovered density the following spring. Hence, N_t+1_/N_t_ is the per-capita population growth rate in absence of migration and birth. It is described by five different models expressing five different hypotheses of how winter mortality changes with density or size in autumn (denoted by X_t_ in the equations):

1. Mortality M is independent of density or size and density only changes with a constant background mortality rate m.

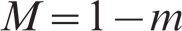
(1)2) Mortality M is linearly density- or size-dependent with the strength given by the mortality rate m.

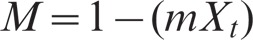
(2)3) Mortality M is linearly density- or size-dependent with the strength given by the mortality rate m_1_ but it has also an independent component m_2_.

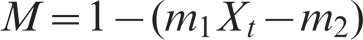
(3)4) Mortality M is non-linearly density- or size-dependent with the strength given by the exponential mortality rate m.

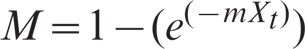
(4)5) Mortality M is non-linearly density- or size-dependent with the strength given by the exponential mortality rate m_1_ but has also an independent component m_2_.
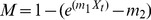
(5)

#### Growth

For each density or size treatment the winter body growth G was calculated as the difference in mean size of log_e_-transformed individual total lengths in spring samples and mean of log_e_-transformed individual total lengths from larvae in the corresponding initial autumn subsamples. Density-dependent winter growth was modelled according to three different hypotheses of how it changes with autumn density N_t_ or size S_t_ (denoted by X in the equations):

1. Constant, density-independent growth g.

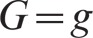
(6)2) Linear density-dependent growth.

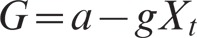
(7)3) Nonlinear density-dependent growth.

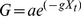
(8)Fitting a van Bertalanffy model to the growth data from Exp. II was unsuccessful: the procedure was numerically unstable and the model did not converge, most likely due to too few data relative to parameter values and/or high variability. Using biomass instead of length obtained from published length-weight regressions gave similar results and did not change the conclusions. However, several published regressions exist that differ sometimes substantially in parameter values. This variability in regressions may reflect population specific relationships and since no such specific data were available for the population used here the analyses were performed with body lengths instead of converted body weights.

A similar analysis as for winter growth was performed for pupation P in Exp. I where P was calculated as number pupae in spring divided by autumn density and was modelled as either density-independent or as changing with density in a linear or exponential fashion.

The models were fitted to the data using linear or non-linear least square regression and were compared to each other with the help of the corrected Aikaike Information Criteria (AICc) computed from each model’s maximum likelihood. For parameters of all models with a difference in AICc smaller than 2 (Δ AICc <2) the 95% confidence intervals were computed using parametric bootstrapping (repeated re-sampling method with replacement; [39]). This model selection procedure avoids the assumption of one single best model describing the data and thus accounts for the uncertainty in model selection [40]. Further, bootstrapping methods are robust and assumption-free; in particular they do not require normality of residuals or equal variances across groups. When bootstrapped 95% confidence intervals do not contain zero the parameters are by definition statistically significantly different from zero at an α-level of 0.05 [39].

To support the analysis of winter growth based on differences in mean body lengths the size distributions in autumn and spring were calculated as probability density functions for each of the twelve density treatments in Exp. I and for each of the four size treatments in Exp. II 95% confidence envelopes for these probability density functions were computed using bootstrapping. Again, where the envelopes do not overlap, size distributions are statistically significant from each other at an α-level of 0.05.

All calculations and analyses were carried out in R [41]. Data for both experiments are available from the Dryad Digital Repository at http://dx.doi.org/10.5061/dryad.

## Results

**Figure 1:**
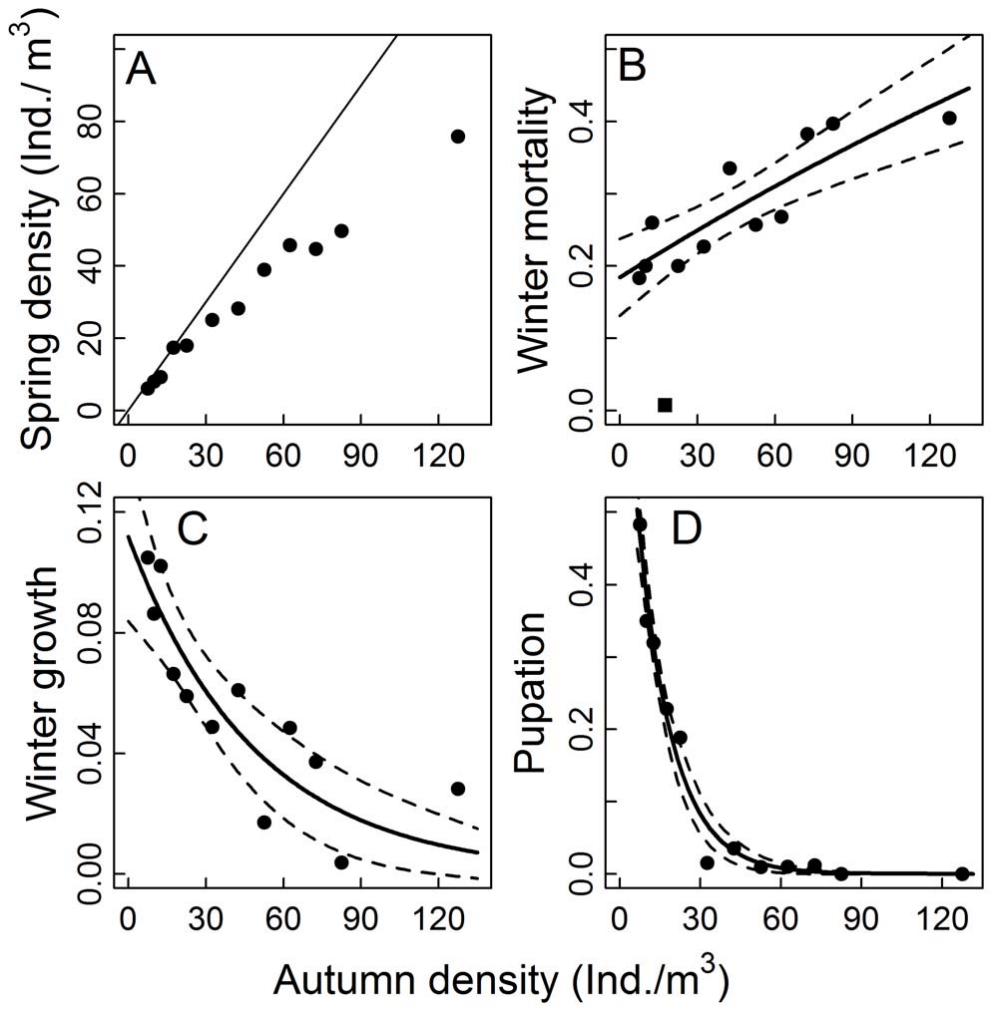
The results of the density dependence experiment (Exp. I). (A) Recovered density in spring, (B) winter mortality calculated as 1- spring density/autumn density, (C) winter growth as spring mean log_e_-transformed total lengths – autumn mean log_e_-transformed total lengths, (D) pupation rate as number of pupae in spring/number of *Chaoborus* larvae in autumn; all as functions of stocked autumn densities. Black symbols are the observed values. The black square in (B) is a statistical outlier that was removed in the analysis. The straight line in (A) represents the 1∶1 ratio of autumn and spring densities. In (B) – (D) solid lines give the fit of the highest ranked model based on the corrected AIC value (see Table 1A–C) and dashed lines give its 95% confidence interval.

**Figure 2:**
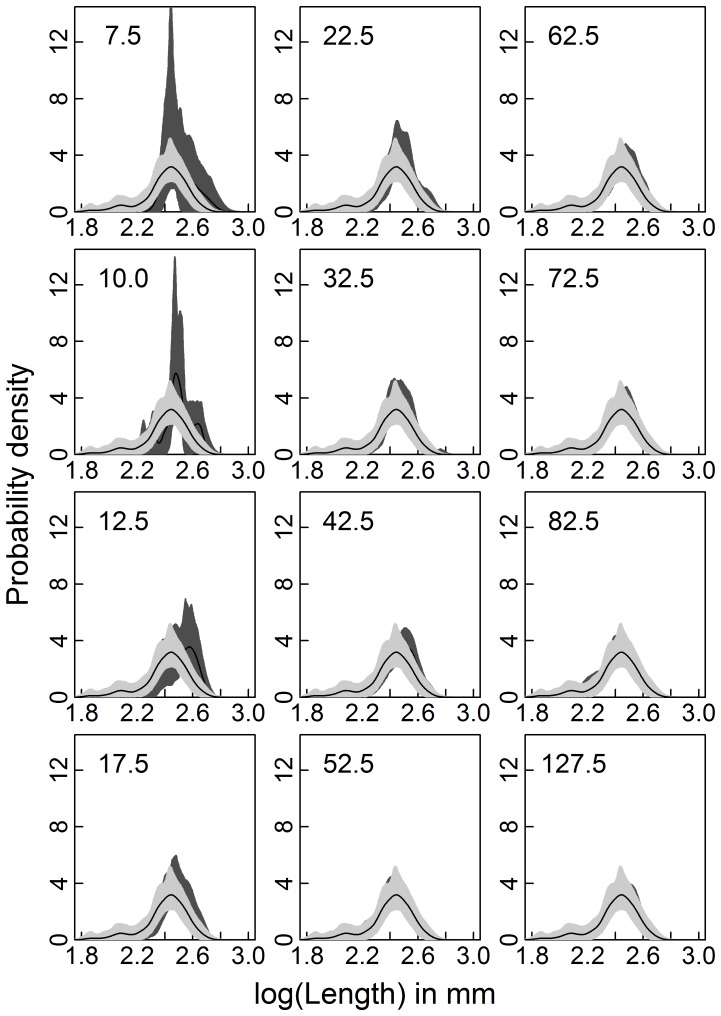
Individual body lengths distributions of *Chaoborus* larvae for the density dependence experiment (Exp. I). Distributions are expressed as probability densities in autumn (light grey) and in spring (dark grey). The lines give the mean probability densities and the shaded areas its bootstrapped 95% confidence interval. The numbers in each panel are the densities (larvae m^−3^) with which enclosures were stocked in autumn.

In Exp. I (density dependence), winter mortality increased exponentially with autumn density but it also had a strong density-independent component (Table 1, Figures 1A, B) so that in the enclosure with the lowest stocked density 80% (74–84; model estimate and its bootstrapped 95% CI) of all *Chaoborus* larvae survived while at the highest density the survival chance was estimated to be only 56% (42–67; Figure 1A). Differences in mean lengths between autumn and spring declined exponentially with autumn density (Table 2, Figure 1C). Since the size frequency distributions shifted to larger sizes (Figure 2) it can be concluded that larvae grew over winter. When autumn density was lowest, the mean spring sizes of larvae was an estimated 12.34 mm (11.95–12.72) and thus 10.09% (6.57–13.46) larger than in autumn, but it reached only 11.31 mm (11.22–11.56) at the highest autumn density, which was only slightly larger (0.83%, 0.09–3.11) than before the winter (Figure 2). The proportion of *Chaoborus* larvae in autumn that had pupated at the termination of the experiment in spring also decreased with autumn density (Table 3, Figure 1D). While at the lowest autumn density nearly half of all larvae stocked in autumn had become pupae (0.47 0.23–0.81) at the end of the experiment, the strong negative density dependence led to a decline of the proportion of pupae to 0.07 (0.01–0.19) already at a stocked autumn density of 32.5 larvae m^−3^.The proportion of pupae continued to go down with further increased autumn larvae density so that at higher densities no pupae were found in enclosures (Figure 1D).

**Table 1:** Model selection details, model parameters and their bootstrapped 95% confidence intervals (in square brackets, R = 1000) for the density dependence experiment (Exp. I); analysis of *winter mortality = *1– spring density/autumn density.

Models fitted	K	X	AICc	Δ AICc	Weight	Ratio	Parameter	
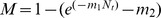	3	11	−30.626	0.000	0.991	1.000	**m_1_** = 0.0023 [0.0015, 0.0033]**m_2_** = 0.1842 [0.1527, 0.2334]	
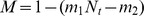	3	11	−20.457	10.169	0.006	165.167	NA	
M = 1– m	2	11	−19.100	11.526	0.003	330.333	NA	
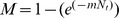	2	11	−13.521	17.105	0.000	Inf	NA	
M = 1– (mN_t_)	2	11	20.655	51.281	0.000	Inf	NA	

N_t_ in equations stands for stocked autumn density.

K = number of degrees of freedom, X = number of data points, AICc = corrected Aikaike Information Criterion, Weight = Aikaike weight, Ratio = evidence ratio.

**Table 2:** Model selection details, model parameters and their bootstrapped 95% confidence intervals (in square brackets, R = 1000) for the density dependence experiment (Exp. I); analysis of *winter growth* = mean log_e_(spring total lengths) - mean log_e_(autumn total lengths).

Models fitted	K	X	AICc	Δ AICc	Weight	Ratio	Parameter
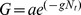	3	12	−58.818	0.000	0.966	1.000	**a** = 0.1120 [0.0830, 0.1380]**g** = 0.0204 [0.0118, 0.0354]
G = a – gN_t_	3	12	−52.066	6.753	0.032	29.273	NA
G = g	2	12	−44.455	14.363	0.001	966.000	NA

N_t_ in equations stands for stocked autumn density.

K = number of degrees of freedom, X = number of data points, AICc = corrected Aikaike Information Criterion, Weight = Aikaike weight, Ratio = evidence ratio.

**Table 3:** Model selection details, model parameters and their bootstrapped 95% confidence intervals (in square brackets, R = 1000) for the density dependence experiment (Exp. I); analysis of *pupation* = pupae/autumn density.

Models fitted	K	X	AICc	Δ AICc	Weight	Ratio	Parameter
P = be^(−pN^_t_^)^	3	12	−47.529	0.000	1.000	1.000	**b** = 0.8306 [0.6211, 1.2551]**p** = 0.0768 [0.0581, 0.1311]
P = b – pN_t_	3	12	−10.537	36.992	0.000	Inf	NA
P = p	2	12	−4.085	43.444	0.000	Inf	NA

N_t_ in equations stands for stocked autumn density.

K = number of degrees of freedom, X = number of data points, AICc = corrected Aikaike Information Criterion, Weight = Aikaike weight, Ratio = evidence ratio.

**Figure 3:**
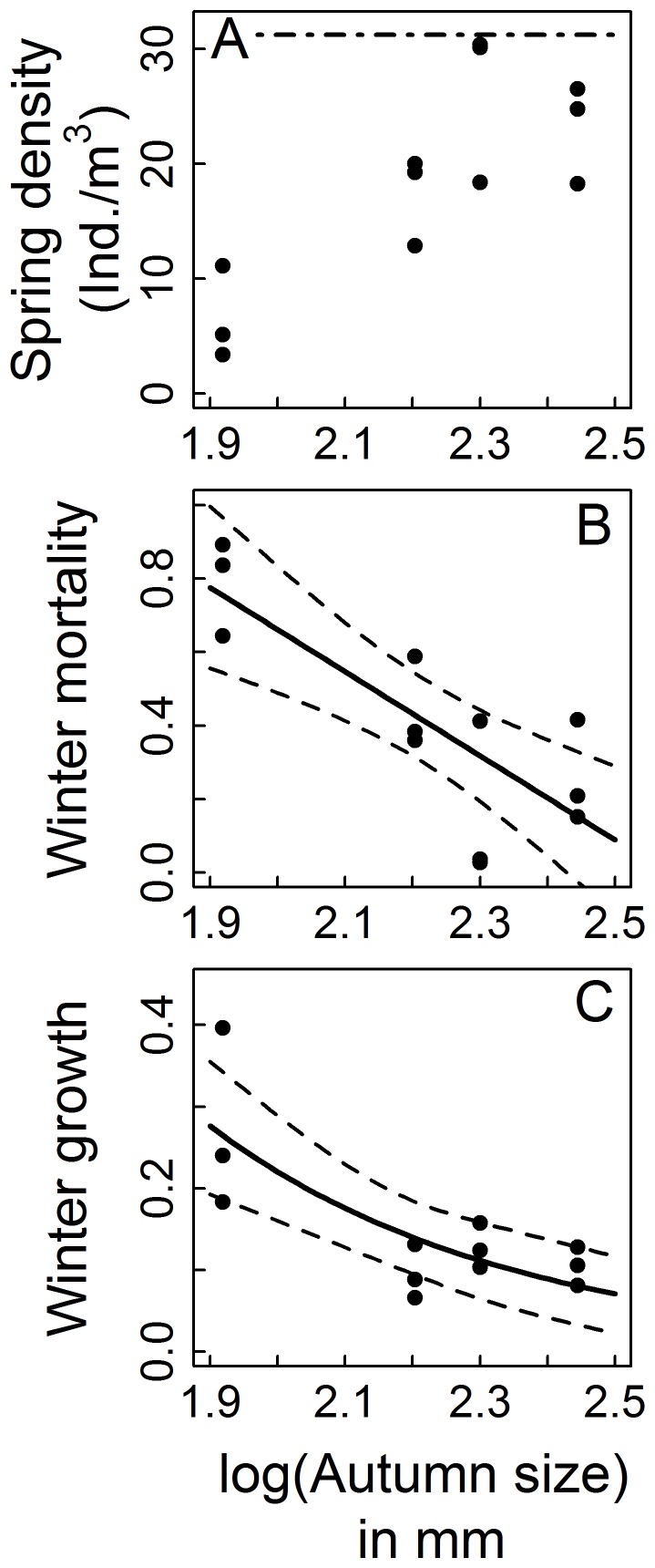
The results of the size dependence experiment (Exp. II). (A) Recovered density in spring, (B) winter mortality = 1- spring density/autumn density, (C) winter growth as spring mean log_e_-transformed total lengths – autumn mean log_e_-transformed total lengths; all as functions of mean autumn body lengths. Black symbols are the observed values. The dotted-dashed horizontal line in (A) represents the spring stocking density of 31.25 larvae m^−3^. In (B)–(C) solid lines give the fit of the highest ranked model based on the corrected AIC value (see Table 2A–B) and dashed lines give its 95% confidence interval.

**Figure 4:**
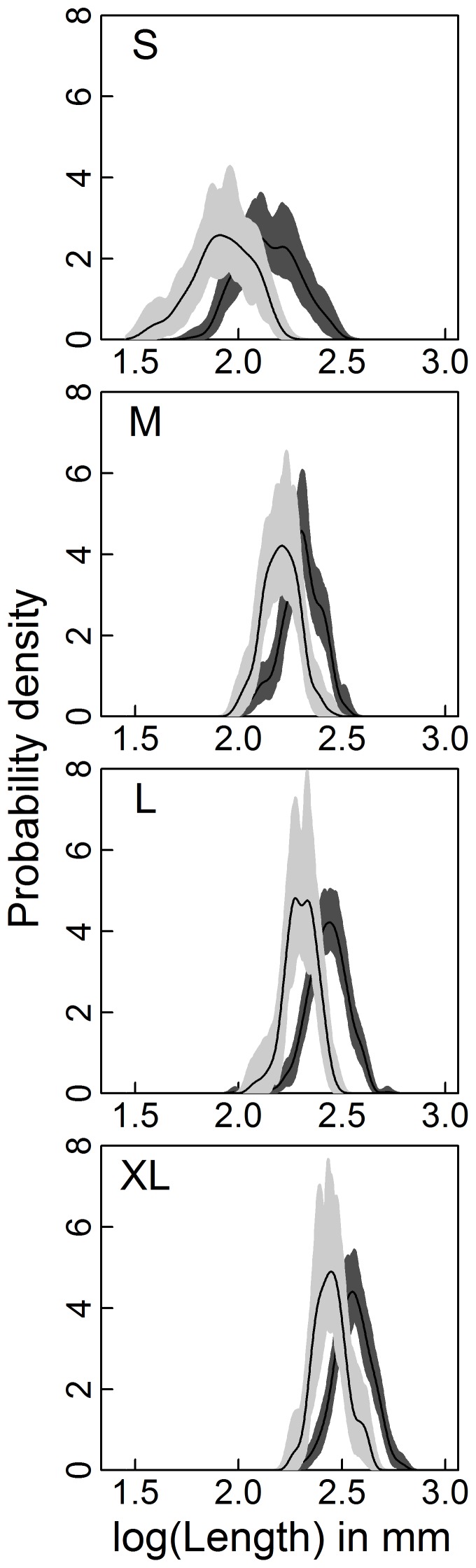
Individual body lengths distributions of *Chaoborus* larvae for the size dependence experiment (Exp. II). Distributions are expressed as probability densities in autumn (light grey) and in spring (dark grey). The lines give the mean probability densities and the shaded areas its bootstrapped 95% confidence interval. The letters in each panel represent the size treatments S, M, L, and XL with which mesocosms were stocked.

In Exp. II (size dependence), winter mortality decreased with total body length but as for density dependence there was a significant background mortality. Linear and non-linear decline remain viable hypotheses for the form of the size-dependent mortality component, at least over the range of body lengths tested here, as AICc values for the two corresponding models were well within two units (Table 4). Here the estimates including their 95% CI from the highest ranked model which assumed linear negative size dependence are given. Due to the size-dependent mortality only 25% (19–44) of the individuals of the smallest size class survived the winter while of those in the largest size class 85% (74–96) of larvae stocked in autumn could be recovered in spring (Figures 3A, B). The seasonal size differences were negatively affected by autumn body lengths (Table 5, Figure 3C), too, and the size distribution shifts to the right indicate that this was due to body growth (Figure 4). Again, linear and non-linear growth functions could not be unambiguously distinguished from each other but here the estimates from the highest ranked model with non-linear negative size dependency of winter growth are given. Larvae in the smallest size class were 27.74% (3.42–85) larger in spring than in autumn and reached a mean length of 8.73 mm (7.04–12.70) whereas larvae in the largest size class were only 8.32% (0.81–25.86) larger in spring than in autumn and reached a mean length of 12.49 mm (11.62–14.51). No pupae were found in any enclosure of this second experiment at the time of its termination. The percentage of instars III declined between autumn and spring from 70% to 3.5% when enclosures were stocked with the smallest size class and from 18% to 0.7% in the “Medium” size treatment.

**Table 4:** Model selection details, model parameters and their bootstrapped 95% confidence intervals (in square brackets, R = 1000) for the size dependence experiment (Exp. II); analysis of *winter mortality* = 1– spring density/autumn density.

Models fitted	K	N	AICc	Δ AICc	Weight	Ratio	Parameter
M = 1–(m_1_S_t_ – m_2_)	3	12	−0.616	0.000	0.545	1.000	**m_1_** = 1.1447 [0.5669, 1.5746]**m_2_** = 1.9509 [0.6456, 2.8956]
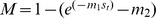	3	12	−0.150	0.466	0.420	1.298	**m_1_** = −0.4359 [−0.5289, −0.2967]**m_2_** = 2.0509 [1.3309, 2.5678]
M = 1–(mS_t_)	2	12	5.513	6.129	0.026	20.961	NA
M = 1–m	2	12	8.258	8.874	0.006	90.833	NA
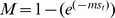	2	12	10.155	10.771	0.02	265.000	NA

S_t_ in equations stands for stocked mean autumn body size.

K = number of degrees of freedom, X = number of data points, AICc = corrected Aikaike Information Criterion, Weight = Aikaike weight, Ratio = evidence ratio.

**Table 5:** Model selection details, model parameters and their bootstrapped 95% confidence intervals (in square brackets, R = 1000) for the size dependence experiment (Exp. II); analysis of *winter body growth* = mean spring total length/mean autumn total length – S_t_ in equations stands for stocked mean autumn body size.

Models fitted	K	N	AICc	Δ AICc	Weight	Ratio	Parameter
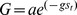	3	12	−26.049	0.000	0.708	1.000	**a** = 20.264 [9.5610, 31.1024]**g** = 2.2636 [2.0066, 2.8968]
G = a – gS_t_	3	12	−24.121	1.928	0.270	2.622	**a** = 0.87 [0.3448, 1.5701]**g** = 0.3251 [0.0924, 0.6211]
G = g	2	12	−19.148	6.901	3 0.022	32.182	NA

K = number of degrees of freedom, X = number of data points, AICc = corrected Aikaike Information Criterion, Weight = Aikaike weight, Ratio = evidence ratio.

## Discussion

The two experiments revealed that growth and mortality in overwintering late instar larvae of *Chaoborus flavicans* in boreal lakes and ponds can be dependent on autumn density and on autumn body size in a generally non-linear fashion, and the analysis also revealed a density- or size-independent background overwinter mortality.

The mechanisms behind the relationships are likely due to resource availability. Predation can be excluded as no fish or other large invertebrate predators were present in the enclosures and while size-specific cannibalism can occur in some *Chaoborus* species [33], the autumn size distributions used were quite narrow, especially in the size dependence experiment, suggesting a minor impact of cannibalism. Instead it seems that food and energy limitation caused the observed patterns. At high densities there was then not enough prey for all larvae to survive and small larvae did not have enough energy reserves to avoid starvation. This interpretation further suggests that summer conditions may influence *Chaoborus* winter survival via summer growth and energy accumulation as has been demonstrated in fish [7], [10]. Still, some of the larvae in the smallest size treatment survived and even grew substantially; these are probably those individuals that did not starve until late winter when production started again with the increasing light intensity under the ice [2]. This interpretation is consistent with data from a Canadian lake where IV-instar *C. flavicans* larvae gained biomass between February and May but not between November and February [36] and with reported higher spring respiration rates of *C. flavicans* IV-instars compared to autumn respiration rates [35]. Clearly, *C. flavicans* larvae are metabolically active during winter. It is however unclear to what degree larvae moult during winter. Given the observed positive winter growth and size-dependent mortality, the virtual absence of III-instars in spring in those treatments receiving some (Small and Medium) is probably due to a mixture of low survival rates and moulting.

It is also interesting to think about whether the higher growth rate of the small surviving larvae will be enough to compensate the size disadvantage in autumn so that they can pupate and how fecund the adults would be that emerge from these pupae. Reduced fecundity of adults emerging from smaller overwintering larvae may be one mechanism with which size- and density-dependent winter processes can affect *Chaoborus* populations in summer. However, no pupae were found in the size dependence experiment when terminated only two to three days after ice break, either because of the autumn density used (Figure 1D) or because of low temperature-dependent pupation in *Chaoborus* [42], [43] in the cold and late spring of 2012. Due to the fixed termination day (pupation was not part of the original research hypotheses) also for the density dependence experiment it remains unknown whether the observed density dependence in the proportion of pupae would translate into density dependence in the total number of larvae pupating in spring or whether it is the pupation rate, time of emergence or adult fecundity that are density-dependent.

The high mortality of small larvae is in line with the univoltine life cycle of northern *Chaoborus* species [36], [37], [44]. There are some data suggesting that sometimes a second cohort emerges in late summer [36] (A. Schröder unpublished data) but such a life history seems to be beneficial only when environmental conditions are conducive for these larvae to reach large sizes by beginning of winter. The regional differences and local adaptations in *C. flavicans* ecological requirements, life history and physiology have not been systematically studied but in general the species seems to be predominantly univoltine at least between North-east mainland Europe (53°00′North, 13°33′East) [44] and Northern Scandinavia (64°29′North, 19°26′East) [22]. In other regions, for example in a eutrophic pond in South Japan (36°20′North, 140°07′East) at least two generations per year can occur [45].

Density and size dependence in winter processes may also establish or influence temporal fluctuations in population densities, especially when non-linear as observed here. In *Chaoborus* with its non-overlapping generations and annual succession of distinct larval, pupal and adult stages winter effects are readily transferred across life history stages and thus seasons. For example, strong density dependence in winter processes may carry over to low densities in summer which allows high winter survival or growth the next winter, possibly leading to regular population cycles. Inter-annual density fluctuations have indeed been observed in several *Chaoborus* populations of varying species though not necessarily cyclic dynamics as time series were usually too short [22], [44], [46], [47]. So far, density differences in *Chaoborus* populations have usually been linked to variation in abiotic factors and interspecific interactions (reviewed in [48]) or to indirect food web effects of trophic interactions and of environmental change (e.g. competition between rotifers and cladocerans [38], lower predation by invertebrates intolerant to low pH after acidification [49], nutrient loading [27], [28]). The results presented here suggest that also density dependence in vital rates may contribute to long-term fluctuations especially when fluctuations appear to be regular and when non-linear density effects are delayed across life history stages [50]. However, to fully understand long-term population dynamics of *Chaoborus* more information is required on other processes and their dependence on body size and density. In fact, the strongest density dependence was here found in pupation but without data on growth, moulting and survival over summer and adult fecundity and survival in spring, the relative importance of different processes and how *Chaoborus* populations are regulated will remain unclear.

Overall, the results imply that winter processes can have important fitness and dynamical consequences for *Chaoborus* and possibly other pelagic invertebrate consumers. The work presented here fills an important gap in *Chaoborus* ecology and will thus lead to a better understanding of the population dynamics of this major invertebrate consumer, including its impacts on lake and pond food webs within and across different seasons. The work also underlines the notion that winters are not a time of dormancy for boreal and temperate freshwater ecosystems and their invertebrate consumers.
